# Prof. Paul F. Eve

**Published:** 1851-10

**Authors:** 


*Prof. Paul F. Eve.*—Prof. Eve has accepted the Chair of Surgery in the Medical Institution of Nashville, Tenn.

